# Aging of stimulus-driven and goal-directed attentional processes in young immigrants with long-term high altitude exposure in Tibet: An ERP study

**DOI:** 10.1038/s41598-018-34706-y

**Published:** 2018-11-27

**Authors:** Hailin Ma, Xiaoyan Huang, Ming Liu, Huifang Ma, Delong Zhang

**Affiliations:** 1 Plateau Brain Science Research Center, South China Normal University/Tibet University, Guangzhou, 510631/Lhasa 850012 China; 2 Center for the Study of Applied Psychology, Key Laboratory of Mental Health and Cognitive Science of Guangdong Province, School of Psychology, South China Normal University, Guangzhou, China; 3 Institute for Brain Research and Rehabilitation, South China Normal University, Guangzhou, 510631 China; 4 College of Management, Tianjin University, Tianjin, China

## Abstract

High altitude (HA) exposure reduces the behavioral response to visual attention and the neural basis is still largely unclear. The present study explored the stimulus-driven and goal-directed factors that are hidden within this attentional behavior impairment via a visual search paradigm in young immigrants in Tibet by recording event-related potential (ERPs). We found that HA explosure significantly slowed the stimulus-driven behaviors instead of the goal-directed behaviors. Furthermore, the P1, N1, and P3 amplitudes collectively indicated the poor efficiency of entire attention behaviors, in which the P3 magnitude of resources allocation was negatively correlated with the attentional behavior response. And the P3 scalp distribution suggested a compensation for insufficient resources of sensory processing only in the goal-directed behaviors. Together, the present study made the point on how stimulus-driven and goal-directed attentional behaviors changed as a result of chronic HA environment exposure, which is similar to aging.

## Introduction

Many studies have indicated behavioral changes under the HA environment, especially motor deterioration and reaction slowness^1–4^. Note that human behaviors generally manifest as the automatic response to external stimuli (stimulus-driven behaviors) and the expression of internal volition (goal-directed behaviors) in the surrounding world^5–8^. And these two points are particularly reflected in attention behaviors. As we noted, visual attention behaviors, such as detecting rapidly threatening stimuli or foraging for your favorite cookies in a crowded supermarket shelves, are very important for the survival of human beings^9^ and are susceptible to the HA exposure^3,10,11^. However, it is still largely unclear how these two points changed with the injure of visual attention behaviors under the HA exposure.

Plateau sections (2,500 m above sea level) holding more than 140 million permanent population are mainly distributed in North, Central, and South America, East Africa, and Asia^12^. In China, the Qinghai-Tibetan Plateau is a typical plateau section with hypoxia, low pressure, dry climate, strong ultraviolet rays, thin air, and low temperatures. Of note, there are about 12 million permanent population on the Qinghai-Tibetan Plateau (71.14% of them live between 2,500 to 4,500 m) as of 2006 and approximately 6 million lowland immigrants^2,13,14^. Moreover, hundreds of thousands of people in lowland areas travel, learn and work, especially engaging in disaster relief and military activity, to the Tibetan plateau every year^2^. Previous studies have discovered the longer time of staying at HA area, the longer the duration of the attention reduction. It would take long time in recovery after people returned to the low altitude (LA) area^15^. And the higher in the altitude place, the less things people can notice^16^. This declining attention performance is claimed to be caused by changes of the functioning neural systems which support attention processes^17^. In many previous studies, the role of attention behavioral processes including the stimulus-driven behaviors and the goal-directed behaviors was paid more attention, which showed that both were independent^18–21^, suggesting that they had different neural pathways^5,22,23^. However, more recent studies emphasized the relationship of both attention behaviors^24^ in which how the stimulus-driven behaviors competed with the goal-directed behaviors when they mutually coordinated attention behaviors^25^. More details, the stimulus-driven behaviors won the competition^24^, as indicated by more rapid orientation and greater accuracy in the stimulus-driven behaviors^26–28^. Conversely, the stimulus-driven behaviors could be prevented but the goal-directed behaviors could be more prominent^29^. However, the stimulus-driven behaviors and the goal-directed behaviors were less prominent in schizophrenia^30^ and the equilibrium and the integration of two attention processes were deficient in psychopaths^31,32^. The similar consequence was also presented in the elders who performed more poorly than the younger in both attention processes, especially the goal-directed attention^33–35^. By contrast, the goal-directed attention showed an age-equivalent modulation in the visual selective attention^36^. Furthermore, aging goal-directed behaviors showed a weakened activation from occipitotemporal areas but increased contributions of anterior regions^37,38^.

To investigate directly and concretely those two attention processes, we used a visual search paradigm that combined the stimulus-driven attention with the goal-directed attention creatively^26,39^. In this paradigm, a target appeared among 3 distracters in separate stimulus-driven attention in which the distracters and the target were different in both color and orientation so that the target was seen primarily to draw attention automatically, and the goal-directed attention in which the distracters and the target were different only in orientation so that making more effort to find the target. Meanwhile, to better observe the changes of two attention processes under the HA environment, we would use ERPs as a measure owing to their preeminent temporal resolution and topography of ongoing neural activity of behaviors and their sensitivity to reveal processes that are not overt in behavior^40^. For those details that two attention processes evoked an early P1 and N1 component^41–44^ and the goal-directed attention generated a typically larger N1 component and more decreased P3 component than the stimulus-driven attention^21,26,45^, we used the P1, N1 and P3 components to measure this task systematically that was practised on participants containing young inhabitants in the LA area and those who were born and grew up in sea level areas but then migrated to Tibet for a relatively long period. We hypothesized that two attention processes have poor performance in young immigrants in Tibet which can be indexed by changes of the ERP component.

## Results

### Behavioral Results

Table 1 illustrates the mean RTs and accuracy rates of each group. The results of a two-way ANOVA with condition (stimulus-driven attention and goal-directed attention) and group (LA group and HA group) as factors showed that the main effect of condition was significant for mean RTs [*F*(1,56) = 838.83; *p* < 0.001; ${{\rm{\eta }}}_{p}^{2}$ = 0.94], with slower RTs in the goal-directed attention than the stimulus-driven attention. In addition, the result of a one-way ANOVA demonstrated that there was a significant difference between groups in the stimulus-driven attention [*F*(1,56) = 4.40; *p* = 0.04], slower RTs in the HA group than in the LA group. But no significant differences between groups were found in RTs for the goal-directed attention (*p* > 0.05). The two-way ANOVA showed that there was a main effect of condition for accuracy rates [*F*(1,56) = 58.07; *p* < 0.001; ${{\rm{\eta }}}_{p}^{2}$ = 0.51], with more errors in the goal-directed attention than in the stimulus-driven attention. However, no significant differences were found in accuracy rates for either condition between two groups (*p* > 0.05).

**Table 1:** Averaged RTs (ms) and accurate rates (%) and their corresponding standard deviations for each of two attention processes (Stimulus-driven attention; Goal-directed attention) in the HA and the LA groups.

	RTs		Accuracy	
	Stimulus-driven	Goal-directed	Stimulus-driven	Goal-directed
HA group	617.44 ± 134.09	1030.72 ± 182.67	98.69 ± 0.01	96.80 ± 0.02
LA group	558.18 ± 71.92	978.53 ± 128.88	99.01 ± 0.01	96.92 ± 0.02

### ERP Results

**Figure 1:**
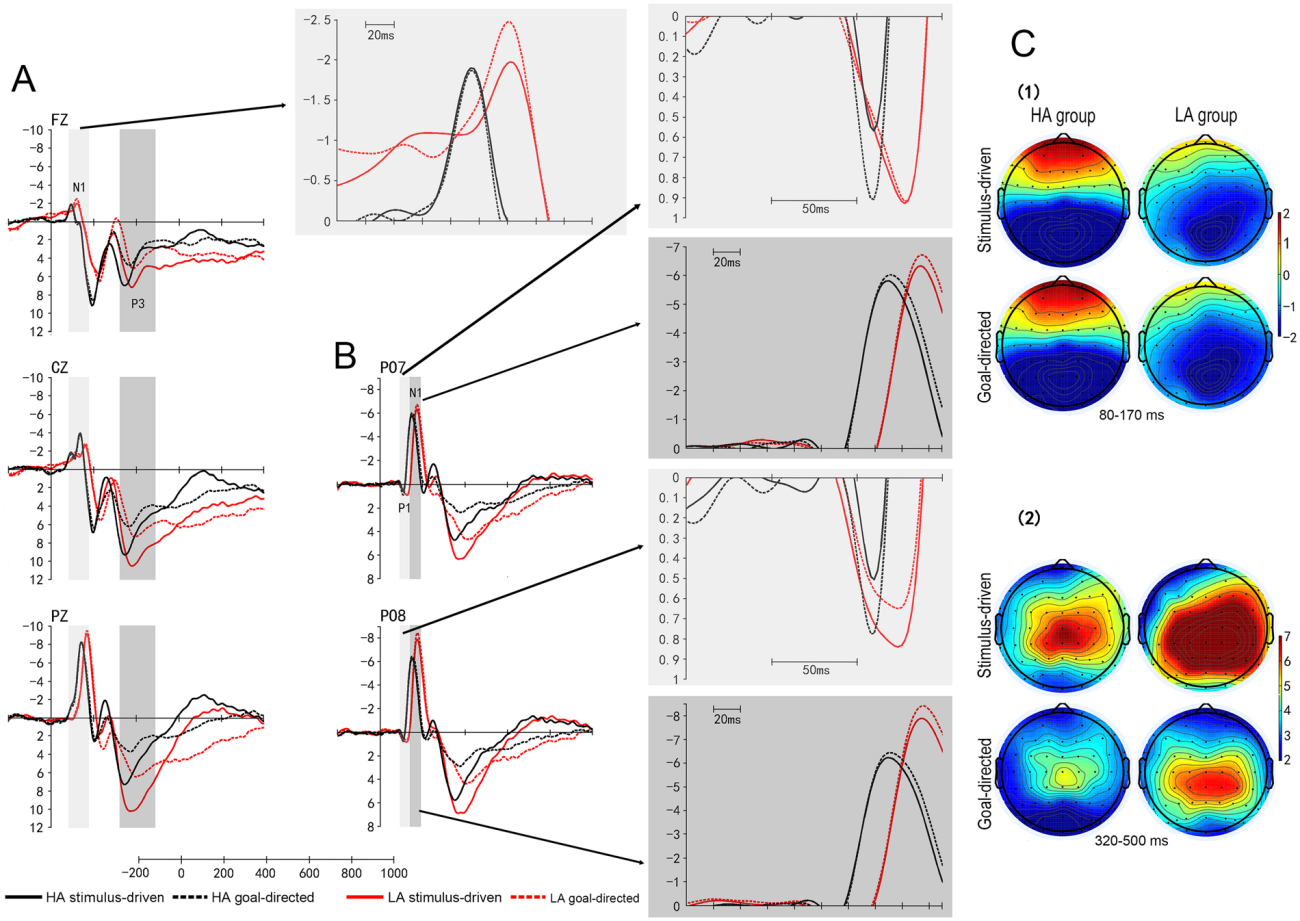
ERP waveforms and topographic map of two groups. (**A**) Averaged ERPs at FZ, CZ, and PZ electrodes for the stimulus-driven attention and the goal-directed attention in the HA group and the LA group. (**B**) Averaged ERPs at PO7 and PO8 electrodes for the stimulus-driven attention and goal-directed attention in the HA group and the LA group. (**C**) (1) Scalp topographies of the mean amplitude within the N1 time-window for the stimulus-driven attention (upper) and the goal-directed attention (bottom) attention in the HA group (left) and the LA group (right). (**C**) (2) Scalp topographies of the mean amplitude within the P3 time-window for the stimulus-driven attention (upper) and the goal-directed attention (bottom) attention in the HA group (left) and the LA group (right).

*P1*. Figure 1B presents the mean change of ERPs at the PO7 and PO8 electrodes for the stimulus-driven attention and the goal-directed attention in either group, and Table 2 summarizes the P1 mean amplitude. A three-way ANOVA was performed with electrode location (PO7 and PO8), condition (stimulus-driven attention and goal-directed attention) and group (LA group and HA group) as factors. There was a main effect of group [*F*(1,56) = 9.84; *p* = 0.003; ${{\rm{\eta }}}_{p}^{2}$ = 0.15], with a smaller P1 amplitude in the HA group than in the LA group. Further the result of a one-way ANOVA found a smaller P1 amplitude in the HA group than in the LA group at PO7 electrode and PO8 electrode of the stimulus-driven attention [PO7: *F*(1,56) = 7.92; *p* = 0.007; ${{\rm{\eta }}}_{p}^{2}$ = 0.12; PO8: *F*(1,56) = 10.25; *p* = 0.002; ${{\rm{\eta }}}_{p}^{2}$= 0.16] and the goal-directed attention [PO7: *F*(1,56) = 5.84; *p* = 0.02; ${{\rm{\eta }}}_{p}^{2}$ = 0.09; PO8: *F*(1,56) = 7.20; *p* = 0.01; ${{\rm{\eta }}}_{p}^{2}$ = 0.11].

**Table 2:** P1, N1 mean amplitudes ( μV) and their corresponding standard deviations at PO7 and PO8 electrodes for each of two attention processes (Stimulus-driven attention; Goal-directed attention) in the HA and the LA groups.

	P1 amplitude		N1 amplitude	
	HA group	LA group	HA group	LA group
**PO7**				
Stimulus-driven	−0.92 ± 2.10	0.67 ± 2.21	−5.56 ± 3.16	−6.22 ± 4.89
Goal-directed	−0.73 ± 2.14	0.65 ± 2.21	−5.79 ± 3.07	−6.59 ± 4.60
**PO8**				
Stimulus-driven	−1.07 ± 2.51	0.70 ± 1.60	−6.00 ± 3.76	−7.62 ± 4.64
Goal-directed	−0.91 ± 2.45	0.52 ± 1.52	−6.21 ± 3.87	−8.42 ± 4.32

*N1*. The N1 mean waveforms in sensory-perceptual regions can be seen in Table 2 and Fig. 1B. In a three-way ANOVA with electrode location (PO7 and PO8), condition (stimulus-driven attention and goal-directed attention) and group (LA group and HA group) as factors, there was only a main effect of condition [*F*(1,56) = 11.33; *p* = 0.001; ${{\rm{\eta }}}_{p}^{2}$ = 0.17], with greater amplitude in the goal-directed attention than in the stimulus-driven attention. Furthermore, the result of a one-way ANOVA that was performed with group (LA group and HA group) as factor for the goal-directed attention showed that there was a group difference at PO8 electrode [*F*(1,56) = 4.23; *p* = 0.04; ${{\rm{\eta }}}_{p}^{2}$ = 0.07], with a smaller amplitude in the HA group than in the LA group. Further, a one-way ANOVA was performed with electrode location (PO7 and PO8) as factor in the stimulus-driven attention and the goal-directed attention for either group. Only in the LA group, N1 amplitude was greater at PO8 electrode than at PO7 electrode in the goal-directed attention [*F*(1,28) = 4.41; *p* = 0.045; ${{\rm{\eta }}}_{p}^{2}$ = 0.14]. Figure 1C(1) further presents the scalp topographies of the mean amplitude within the N1 time window, showing that the hemisphere asymmetry was only found in LA group for two conditions, while the left and the right electrode sides were both activated in the HA group for two conditions.

Figure 1A shows the N1 amplitudes for the stimulus-driven attention and the goal-directed attention at FZ, CZ, and PZ electrodes in either group. Table 3 presents the N1 mean amplitude. A three-way ANOVA with electrode location (FZ, CZ, and PZ), condition (stimulus-driven attention and goal-directed attention) and group (LA group and HA group) as factors demonstrated a main effect of electrode location (FZ, CZ, and PZ) [*F*(1,66) = 153.52; *p* < 0.001; ${{\rm{\eta }}}_{p}^{2}$ = 0.73], with PZ > CZ > FZ. Further, the results of a one-way ANOVA showed that there was a smaller amplitude in the HA group than in the LA group at FZ electrode for either condition [stimulus-driven attention: *F*(1,56) = 5.76; *p* = 0.02;${\,{\rm{\eta }}}_{p}^{2}$ = 0.09; goal-directed attention: *F*(1,56) = 8.65; *p* = 0.005;${\,{\rm{\eta }}}_{p}^{2}$ = 0.13].

**Table 3:** N1 and P3 mean amplitudes ( μV) and their corresponding standard deviations at FZ, CZ, and PZ electrodes for each of two attention processes (Stimulus-driven attention; Goal-directed attention) in the HA and the LA groups.

	FZ		CZ		PZ	
	Stimulus-driven	Goal-directed	Stimulus-driven	Goal-directed	Stimulus-driven	Goal-directed
**N1 amplitude**						
HA group	0.83 ± 2.74	0.87 ± 2.74	−1.68 ± 3.49	−1.59 ± 3.39	−8.06 ± 4.94	−8.07 ± 5.07
LA group	−0.71 ± 2.12	−0.96 ± 1.94	−1.54 ± 2.59	−1.76 ± 2.64	−8.59 ± 5.87	−8.88 ± 6.02
**P3 amplitude**						
HA group	4.37 ± 4.06	3.30 ± 3.12	6.76 ± 4.77	4.91 ± 4.03	4.92 ± 4.21	2.74 ± 3.88
LA group	5.51 ± 4.63	3.47 ± 3.88	8.91 ± 5.55	6.00 ± 4.77	8.67 ± 5.58	5.42 ± 5.53

*P3*. The amplitude of the P3 component is shown in Table 3 and Fig. 1A. The results of condition × electrode × group ANOVA suggested a significant main effect of condition and electrode [condition effect: *F*(1,56) = 62.37; *p* < 0.001; ${{\rm{\eta }}}_{p}^{2}$ = 0.53; electrode effect: *F*(1,80) = 14.86; *p* < 0.001; ${{\rm{\eta }}}_{p}^{2}$= 0.21], with a greater amplitude in the stimulus-driven attention than in the goal-directed attention and CZ > PZ > FZ. And there was a marginality difference between groups [*F*(1,56) = 3.14; *p* = 0.08; ${{\rm{\eta }}}_{p}^{2}$ = 0.05], with a smaller amplitude in the HA group than in the LA group. Furthermore, these was a significant interaction between condition and electrode [*F*(1,78) = 16.18; *p* < 0.001; ${{\rm{\eta }}}_{p}^{2}$ = 0.22] and between electrode and group [*F*(1,80) = 4.02; *p* = 0.03; ${{\rm{\eta }}}_{p}^{2}$ = 0.07]. The results of the former showed that there was a greater amplitude for the stimulus-driven attention compared with the goal-directed attention at FZ, CZ and PZ electrodes, respectively. Meanwhile, the results showed CZ > PZ > FZ in the stimulus-driven attention and CZ > FZ, CZ > PZ in the goal-directed attention. And the results of the later showed a greater amplitude at CZ and PZ electrodes than at FZ electrode in the LA group and a greater amplitude at CZ electrode than at FZ and PZ electrodes in the HA group. Meanwhile, there was a smaller amplitude in the HA group than in the LA group at PZ electrode (*p* = 0.01). Further, the results of a one-way ANOVA showed that there was a smaller amplitude in the HA group than in the LA group at PZ electrode for either condition [stimulus-driven attention: *F*(1,56) = 8.36, *p* = 0.005; ${{\rm{\eta }}}_{p}^{2}$ = 0.13; goal-directed attention: *F*(1,56) = 4.56; *p* = 0.04; ${{\rm{\eta }}}_{p}^{2}$ = 0.08]. Figure 1C(2) shows the scalp topographies of the mean amplitude within the P3 time-window for either group, which are similar to the results of the anterior three-way ANOVA about P3 amplitude for either group. In the left column of Fig. 1C(2), a larger activity appeared at central region for the stimulus-driven attention and at fronto-central region for the goal-directed attention in the HA group, while right column of Fig. 1C(2) generates the tendency of the central-parietal distribution for the stimulus-driven attention and the tendency of the central distribution for the goal-directed attention in the LA group.

### Correlation analyses

Table 4 shows a stepwise regression of RTs and P3 amplitude between two groups. The first model only included the data of the LA group, the final significant predictor was FZ that was related negatively to RTs and accounted for 10% of the variance. The second model only included the data of the HA group, the final significant predictor was CZ that was related negatively to RTs and accounted for 21% of the variance.

**Table 4:** Stepwise regression of the averaged RTs (ms) using P3 amplitudes ( μV) at FZ, CZ, and PZ electrodes as predictors.

Variable	*t*	*p*	Beta	Model *R*^2^
**LA group**				
FZ	−2.48	0.02	−0.31	0.10
CZ	−0.64	0.52	−0.13	
PZ	−0.93	0.36	−0.14	
**HA group**				
FZ	−0.51	0.61	−0.10	0.21
CZ	−3.86	0.00	−0.46	
PZ	−0.72	0.48	−0.13	

## Discussion

The departure point for this study was to explore the performance of the two attentional processes following impaired visual attention behavior in healthy young immigrants under the HA environment in Tibet. We found the difference of RTs in the stimulus-driven attention between groups, longer RTs in the HA group. On this basis, we further discovered cognitive resources of the stimulus-driven attention and goal-directed attention behaviors were insufficient in the HA group and the goal-directed attention had an activation of the anterior region at the late stage in the HA group. Moreover, behavior response and resources allocation exhibited a negative correlation at central region in the HA group. These results provided experimental evidence for the changes of stimulus-driven and goal-directed attention behaviors as a result of the HA exposure.

The behavioral results, in a consistence with previous studies^26,28^, showed that there was a longer RTs and higher error rates in the goal-directed attention than in the stimulus-driven attention, suggesting that the paradigm indeed had distinguished two attention processes. Moreover, the HA group had a longer RTs compared to the LA group only in the stimulus-driven attention, showing the slower processing of the stimulus-driven attention in the HA group.

In this study, we first found that the P1 component showed a great difference between two groups, a smaller posterior P1 amplitude in the HA group compared to the LA group for either attention process. Meanwhile, there was a smaller right posterior N1 only for the goal-directed attention in the HA group than in the LA group. Some studies have indicated that several universal ERP components of the attention contain the parietal-occipital P1 and N1^46–48^ that are indexes of the attentional allocation of the early visual processing involved in alerting and orienting and are enhanced under conditions of the difficult attention tasks^47,49–52^. In our study, this difference between two groups for the P1 component might imply that the early alerting and orienting processing of two attention processes depressed in the HA group. The difference of right posterior N1 only for the goal-directed attention between two groups suggested the subdued expression of the goal-directed attention in the early visual processing. The convergent result of above consequences was presented in the distribution difference of N1 component again. There was a hemispheric difference of the N1 amplitude for the goal-directed attention only in the LA group, with a larger N1 amplitude at the right hemisphere than at the left hemisphere, while there was no difference between the two hemispheres for the goal-directed attention in the HA group. Besides, the distribution of N1 amplitude (Fig. 1C(1)) also showed that only the right posterior sites were active for two attention processes in the LA group, while bilateral posterior sites were active in the HA group for two attention processes. Remarkably, the parietal-occipital N1 component usually shows hemispheric asymmetry, especially displaying the dominance of the right hemisphere in visual-spatial attention tasks^53–57^. This dominance of the right hemisphere about the N1 amplitude only in the LA group was similar to previous studies demonstrating the altitude effect on the dominance of the right hemisphere^2^. And this bilateral activation in the HA group also verified the altitude effect on compensatory mechanism for the left hemisphere in attention again^2^ suggesting that the HA group had to invoke bilateral resources to input more effort for two attention processes due to the lack of mental resources. In frontoparietal networks, there was a parallel result between groups, a smaller N1 amplitude in the HA group than in the LA group at the frontal area for either attention process. Note that the frontal N1 components are larger for the attended locations compared with those unattended^58^ in line with the sensory gain control mechanism in which the amount of attention distribution determines the magnitude of the evoked neural activity^59^. So the difference on the frontal N1 between groups also indicates the insufficient amount of attention allocation to the two attention processes in the HA group compared with the LA group.

This performance of decreased mental resources to two attention processes was also reflected in the later P3 component, whose scalp distributions typically located at parietal region^60^. The P3 component is regarded as an index of the allocation of attentional resources in the later conscious processing^37,61–63^ that involves the evaluation processes of stimulus meaning^49,61^. The present result showed a greater P3 amplitude in the stimulus-driven attention than in the goal-directed attention according with previous studies which discovered that the evaluation of novel or salient stimuli, such as the bottom-up condition^26^, pop-out arrays^64,65^ and negative pictures^66^, evoked a larger P3 amplitude because these stimuli had greater information value to attract more interests in survival surroundings. This result therefore suggested that the shorter RTs observed in the stimulus-driven attention than the goal-directed attention might owe much to more allocation of resources to evaluate the stimuli. Compared to the LA group, there was a smaller amplitude in the HA group at the parietal area for either attention process. This result made clear that the HA group might have insufficient resources sequentially so that put less into the evaluation of information meaning for the two attention processes, in accord with previous normal aging studies finding that the parietal P3 amplitude decrease with age^37,67,68^.

These results of P1, N1 and P3 component suggested that in the two attention processes, young immigrants with long-term high altitude exposure performed more poorly than young inhabitants in the LA area, which was consistent with the age-related decrease of the two attention processes^26,34,35^. In addition, the two subprocedures of attention had different performance on the P3 scalp distribution between two groups, as shown in Fig. 1C(2), a central-parietal P3 for the stimulus-driven attention in the LA group, whereas a maximum central P3 for the stimulus-driven attention in the HA group. Previous studies have consistently reported that a parietal maximum P3 component amplitude appears in easy tasks, whereas central maximum P3 component amplitude appears in difficult tasks^69^. These implied that the stimulus-driven attention was difficult for the HA group because of the shortage of mental resources. For the goal-directed attention, there was a central tendency P3 in the LA group, whereas a fronto-central P3 in the HA group, suggesting a more anterior activation distribution and a underactivation of parieto-occipital region in the HA group. These results were in concordance with the aging findings that frontal regions were more involved in the goal-directed attention than posterior, sensory regions, in aging^26^. Universally, some studies observed a underactivation due to brain regions damage in aging^70–72^ that suggested frequently worse performance in the memory, cognitive control and executive control behaviors^73^. Reversely, an unexpected pattern started to appear which showed the activation of brain regions in the older adults, Alzheimer’s disease patients and the mild cognitive impairment patients^73^, especially the frontal area related with the executive control of attention to the environment, suggesting different strategies to compensate for poorer effects of brain damage in higher-level processing^73,74^. Further, a region worked harder resulting in activation due to declining input it received, indicating a compensation for descending processing elsewhere^73,74^. In some studies, more greater prefrontal activity had an adjoint underactivation of occipitotemporal areas in the elder, supporting the view that strategic processes mediated by the prefrontal regions compensated for inefficient sensory processing in the visual pathway with age to reach age-equivalent performance^75–78^. It was important to note that the present study found homologous result in the goal-directed attention of the HA group and there was a smaller posterior N1 and P3 in the HA group than the LA group. By above account, we speculated that the HA group industriously activated more anterior region to compensate for the declining efficiency of sensory processing regions so that the HA group could perform successfully the task of the goal-directed attention and had equivalent performance compared with the LA group, as evidenced by non-significant RTs in the goal-directed attention between two groups. This conformed with other aging studies of attention that activation of anterior region reflected an improved performance in the elders^26,79,80^. Meanwhile, those results declared the separation of two attention processes under deficient resources due to HA environment, a compensation to insufficient resources of posterior area only in the goal-directed attention although insufficient resources in two attention processes, that might state people was more inclined to explore expression of internal volition under HA environment, such as meditation behavior.

Our findings were consistent with Kahneman’s limited-capacity model of attention^81^. It was believed that the total amount of attention resources is limited. But if the sum of resources that the two tasks required is no more than the total number of psychological resources allocated at the same time, it is possible to operate both tasks at the same time. If not, two tasks would interfere with each other, thereby affected effective execution of both tasks. We thought that cognitive resources were insufficient in the HA group similar to the normal aging^37^, so that the two attention processes might be out of control, which mainly manifested as the impairment of two attention processes and compensation for only goal-directed attention. Further, we found that there were certain relationships that supported by evidence from the results of stepwise regression between the amount of resources allocation that this limitation generated and the performance of attention behaviors. The results showed that this correlation was negative between P3 amplitude and RTs in both groups, indicating that the decreasing P3 amplitude was associated with longer RTs, that is, the diminishing resource was associated with the slower responses. In addition, the results of within-group stepwise regression manifested that the relationship between the P3 amplitude and RTs had a different performance between two groups. Significant predictor was FZ in the LA group and significant predictor was CZ in the HA group, indicating that the LA group with diminishing resource of the frontal area exhibited slower responses and the HA group with diminishing resource of the central area exhibited slower responses.

There were some considerations to interpret the present findings. First, although the difference on ERP components indicated the neural mechanism of the declining attention behavior had some changes under long-term HA exposure, the related results should still be interpreted cautiously, because they may be influenced by some social factors (e.g. economic status, education level or culture)^82^. However, we suggest that hypoxia is leading cause of observed changes as this is most important factor of the HA environment. Although the brain accounts for only about 2% of the body’s weight, the brain’s oxygen consumption is up to 20% of the total^83–85^. Therefore, the central nervous system is particularly sensitive to hypoxia, which can cause changes in the brain function and neurons^86^. Previous researches in which inhabitants in the LA area accepted the acute exposure to hypoxia or simulated hypoxia conditions or moderate hypoxia (under the condition of 8 hours hypoxia) found that hypoxia has an effect on attention behavior progressing^10,87^ and executive function^88^. Obviously, hypoxia affect the behavior most directly and most quickly. So we need to find a way to further prove it in the future, although it is not easy. Second, the expression of goal-directed attention exposed to the HA environment need to be considered. Theory of environmental stress believed that various factors in the environment could cause people’s stress response and the view of molecular medicine also declared that the brain tissue created stress response when people exposed to a deleterious environment^89^. Via a coordinated stress response, the brain could avoid or decrease neurologic damage^90^. This indicated that the brain had the potential capacity of self-protection, such as endogenous neuroprotection, in which the brain could confront these threats of hypoxia or hypothermia^91,92^. Connecting with the fact that the goal-directed activity was main aspect of brain function^84^, we speculated that our finding about the preemptive compensation on the goal-directed attention might reflect a mechanism of cerebral protection under the HA environment. Third, these results did not indicate the essence of the present observation reflecting the deficit of the attention function or the adaption of the attention function in the high altitude exposure which is what we need do in the future. Fourth, because attention ability was a very important part of cognitive function, the present study that focused on the altitude effect on attention behavior reflected broadwise the changes of cognitive function, but it must be admitted that the HA environment might affect all sorts of aspects of cognitive function. So the follow-up studies need further investigate the interrelation about various aspects of cognitive function including perception, memory, decision-making and so on. Finally, the cross-sectional nature of the study also is a limitation. Note that this issue about effects of HA environment exposure on brain function is just getting started, so we have used the cross-sectional study to explore this issue at this stage. Certainly, the dynamic longitudinal study is an important research direction and we will do it in the future.

In conclusion, the present study found different expression of two attention behaviors under the HA environment in Tibet versus LA environment at sea level. The cognitive resources of entire attention behaviors were inadequate and this magnitude of resources allocation and behavior response were negatively correlated. The stimulus-driven behaviors and the goal-directed behaviors performed more poorly and the goal-directed behaviors had a compensation of anterior region for descending efficiency of sensory processing as a result of the HA exposure at later stage.

## Methods

### Participants

A total of 58 healthy, right-handed college students (18–25 years old) were recruited for the present study. All of these participants belong to Han race, in which 29 students from Tibet University were defined as HA group, and the rest from colleges and universities in Guangzhou were categorized into LA group. The participants of the HA group had lived in HA (3,650 m) for 3 years and more, but were born and grew up in LA areas (<1,000 m). The participants of the LA group had lived in LA areas all the time. The Raven Intelligence Test^93^ and basic information questionnaire including the scores on the college entrance examination, sleep, and the time of staying at the plateau were measured before the experiment, and there was no significant difference between the two groups in their scores on the Raven Intelligence Test (*p* > 0.05), minimizing the likelihood that any group differences could be explained by intelligence-related factors (see Table 5). All the participants had normal or corrected-to-normal vision with no prior neurological problems. None of them were taking any psychotropic, neurological, or psychiatric medications at the time of testing. The informed consent was obtained for each participant, and the experiment was commanded in accordance with the Declaration of Helsinki and was approved by the Ethics Committee of the Institute of Psychology, South China Normal University.

**Table 5:** The demographic data of the HA and LA groups.

		M ± SD	*p*
CEE	**HA**	505.41 ± 24.98	0.26
	**LA**	521.86 ± 74.51	
RIT	**HA**	46.45 ± 9.26	0.13
	**LA**	50.14 ± 8.87	
PSQI	**HA**	5.32 ± 2.65	0.82
	**LA**	5.15 ± 2.64	
Time	**HA**	4.07 ± 0.70	.000
	**LA**	0.00 ± 0.00	

Note: CEE, the scores on the college entrance Examination; RIT, the scores of the Raven Intelligence Test; PSQI, the scores of sleep; Time, the time of staying at the plateau.

### Stimuli and procedure

The visual stimuli were the 16 particular triangles which had different colors (red or green) and orientations (45°, 90°, 135°, 180°, 225°, 270°, 315°, and 360° from the vertical meridian). They were combined into “stimulus-driven attention” and “goal-directed attention” conditions which were consisted of 4 triangles respectively whose positions were distributed in the 4 quadrants resulting in stimuli subtending a total 5.34° of visual angle from the fixation cross and which consisted of the target and 3 disturbed items. In the “stimulus-driven attention” condition, 3 disturbed triangles were differed from the target in color and orientation, which generated target stimulus to be so salient that could grab it easily. In the “goal-directed attention” condition, 3 disturbed triangles were differed from the target only in orientation, which caused the target stimulus to be so imperceptible in the scene that should make a special effort to search it. All conditions were randomized and equal, and the targets appeared randomly in the left visual field or right visual field.

**Figure 2:**
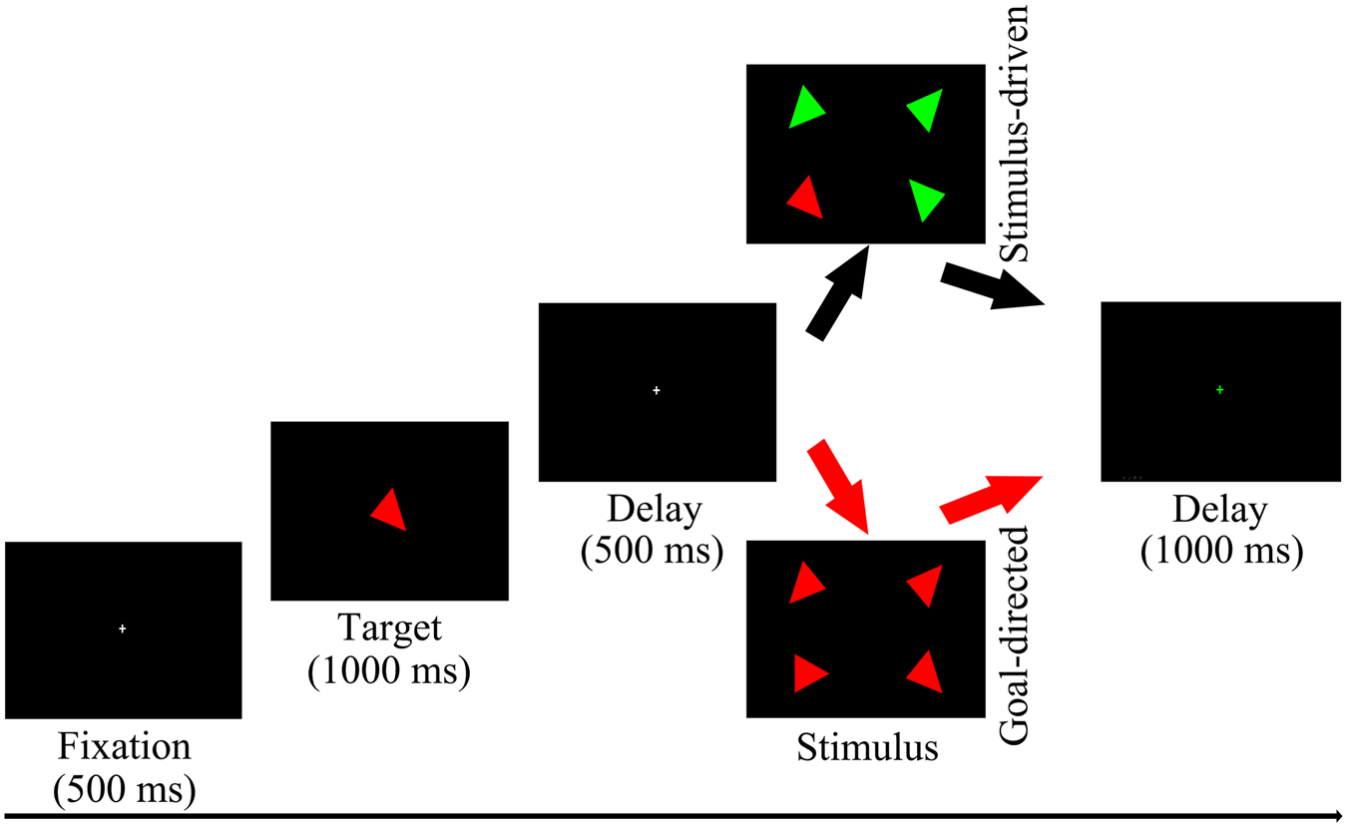
Stimulus sequence of the visual search paradigm.

Figure 2 illustrates an example of the stimulus sequence. A trial begin with a 500 ms fixation cross, followed by a target triangle which had a specific color and orientation and was presented for 1000 ms. Then 500 ms delay screen with a fixation cross which maintained subjects’ central fixation, and followed by the “stimulus-driven attention” or the “goal-directed attention”. Participants were asked to press “F” when targets appeared on the left side of the screen or “J” when targets were on the right side as quickly and accurately as possible, regardless of the vertical position. If no response was made, the array remained on the screen until the participant responded, followed by a 1000 ms green fixation cross to signal the end of the trial. Participants sat in a dimly-lit and quiet room to perform this visual search paradigm which was presented against a black background on a 19-inch computer monitor at a distance of 110 cm. They should complete 12 blocks and each block had 32 trials, resulting in 192 trials in each condition. Sufficient practice was provided to make sure that all participants’ accuracy rate could reach more than 80% before starting the experiment. Extra practice blocks were given as required until participants were able to reach a mean accuracy of 80% in the task. After each block, participants could have a rest.

The stimuli were presented and the behavioral data were collected and analyzed via *E*-Prime software (Version 2.0, Psychology Software Tools, Pittsburgh, PA).

### ERP Recordings and Data Analysis

Brain electrical activity was recorded via 64-channel electrode cap (10/20 system) using Ag/AgCl electrodes in Neuroscan Curry software (version 7.8), with an on-line reference to the middle of the CZ and CPZ locations. Simultaneously, six additional electrodes were recorded including M1 and M2 (because off-line re-reference was the average of the left and right mastoid) and four electrooculogram (EOG). EOG were recorded by placing one electrode above and below the left eye to measure vertical electrooculogram (VEOG) and the outer canthi of both eyes to record horizontal electrooculogram (HEOG), respectively. Ultimately, EEG and EOG were continuously recorded at a sampling rate of 500 Hz, applying less than 5 kΩ electrode impedances.

Trials contaminated by body movements, eye movements, and muscle activity, were rejected offline, with a criterion of ±100 μV. Only trials with correct responses were averaged and segmented into stimulus-locked ERP segments, from 200 ms (baseline) prior to the appearance of stimuli to 1000 ms afterwards, in which the ERP was digitally filtered with a 0.1 Hz–30 Hz band pass filter. Following these analyses, each participant averagely retained about 163 trials for the stimulus-driven attention and 162 trials for the goal-directed attention in the LA group, and retained about 162 trials for the stimulus-driven attention and 161 trials for the goal-directed attention in the HA group.

In stimulus-locked ERP segments, experimental condition elicited clear P1, N1 and P3 components in either group. To reduce the effect of noise, we measured the mean voltage of every component over a time window in which we could find a peak amplitude according to Luck’s recommendation^40^. To observe the early perceptual processing in the stimulus-driven attention and the goal-directed attention for either group, P1 component and N1 component were measured at the PO7 and PO8 electrodes in the 100 ms − 135 ms and 140 ms − 185 ms time window, respectively. In addition, considering close connections between the frontoparietal networks and two attention processes^94–97^, three electrodes (FZ, CZ, and PZ) were selected for N1 and P3 components data analysis in the 80 ms − 170 ms and 320 ms − 500 ms time windows, respectively, to compare the changes of frontoparietal processing in the stimulus-driven attention and the goal-directed attention for either group.

All data were analyzed with SPSS (SPSS, Inc., Chicago). ANOVA was performed on mean RTs for correct responses, accuracy rates and ERP data, whose statistically significance level was set at 0.05 and degrees of freedom of the *F* ratios were corrected according to the Greenhouse-Geisser method. Besides, in order to understand the relation between behavioral expression and resources allocation, a stepwise regression analysis was performed to explore the relationship between dependent variable RTs and predictor variables P3 mean amplitudes at FZ, CZ, and PZ electrodes.
